# Supplementary material for: Probiotics Treatment Improves Hippocampal Dependent Cognition in a Rodent Model of Parkinson’s Disease
Source: Microorganisms. 2020 Oct 27;8(11):1661. doi: 10.3390/microorganisms8111661 (PMC7692862; doi:10.3390/microorganisms8111661)
Supplement: Supplementary file 1 [file microorganisms-08-01661-s001.pdf]

Supplementary Materials

Apparatus for novel object and place recognition tasks

Animals were tested in a 60cm^3 arena with black Perspex walls and a clear Perspex floor with a grey board underneath. The flooring displayed a grid of 16 squares (13.5 cm × 13.5 cm) outlined with black stripes (1 cm thick). Three plastic objects (height ranging from 15.5 cm to 23 cm) were used as stimuli in the novel object recognition/place tasks: a Gatorade™ bottle with no wrapper filled with pink Gatorade™, a Lipton Ice Tea™ bottle with the wrapper and filled with Lipton Ice Tea™ diluted to 75%, and a toy figure of Homer Simpson™. A video camera was placed above the arena in order to record the rats' behaviours. The illumination in the arena was 800 lux.

Apparatus for Elevated plus maze

A wooden elevated-plus maze (EPM) was elevated at 51 cm above the floor. There were two open arms (10 cm wide × 50 cm long with a 1 cm raised edge) and two closed arms (10 cm wide × 50 cm long with 39 cm high walls). The central platform (10 x 10 cm) allowed the animal to travel between open and closed arms of the platform. A video camera was mounted above the maze. The illumination at the open arm was 400 lux.

Novel object recognition and place recognition tasks.

The effect on cognitive function was assessed using novel object recognition and place recognition tasks. The distinction between these tests is either a novel object or the same object moved in a new place. Different brain regions are recruited for novel object and place recognition [56,57]. Spatial cognition is dependent on the hippocampus whereas the novel object recognition task is hippocampal independent [56,57]. These cognitive tests can be applied to test short-term memory, where memory retention for object or place is assessed after 5mins. Short term memory was tested 9 weeks post-surgery (object and place recognition tests were counterbalanced).

The procedures for the novel object recognition and place recognition tasks were adapted from [56 57]. Rats received 2 days of habituation to an empty arena for 10 min per day. Animals were pre-exposed to the arena and allowed to freely explore for 10 min. The arena was cleaned with 70% ethanol between each rat. No objects were present in the arena during habituation. Following habituation to the arena, animals were familiarized to the objects and location and then tested over 4 consecutive days, with the first two days on the either the object or place task and second two days on the other task in a counterbalanced manner. Both tasks began with a familiarization phase. For the familiarization phase, two identical objects were placed in the arena. The animal was allowed to freely explore the arena and objects for 5 min and the time spent with each object was recorded. The animal was then returned to its home cage. An animal was considered to be "exploring" the object if its nose was touching the object and it was actively interacting with the object. The objects and the arena were cleaned with 70% ethanol while the rat was in its home cage.

Analysis for mobility

Mobility was assessed in during the EPM test, measured by the time mobile and the number of entries into open, closed and center zones during the 300second session. Mobility was defined as movement of limbs. The arm entries were defined as entry of at least two paws into the zone.

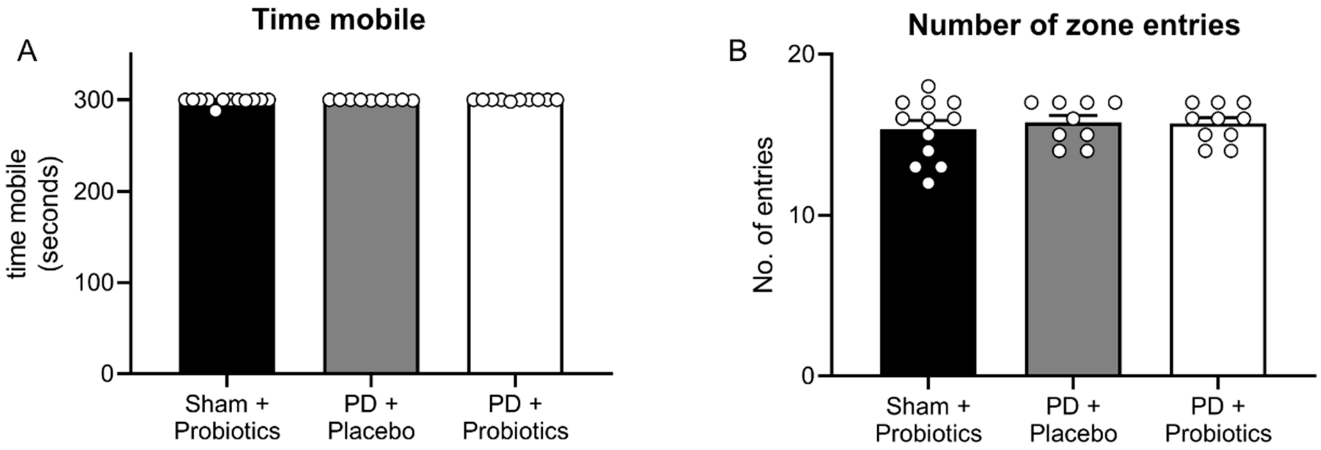

Figure S1. (**A**) Graph of time rats showing mobility assessment during EPM test session. There was no difference in mobility time across all groups. Time mobile (mean ± SEM) in seconds for the sham +probiotics (298.916 ± 0.995), PD + placebo (299.666 ± 0.166) and PD + Probiotics (299.800 ± 0.200), F (2, 30) = 0.516, $p = 0.602$. (**B**) Graph of number of entries in open, closed and centre zones during EPM test session. There was no difference in zone entries across all groups. Number of entries (mean ± SEM) for the sham +probiotics (15.333 ± 0.555), PD + placebo (15.777 ± 0.4333) and PD +Probiotics (15.700 ± 0.366), F (2, 30) = 0.259, $p = 0.774$.
